# Impact of a Structured Social Skills Training Program on Adolescents and Young Adults with Level 1 Autism

**DOI:** 10.3390/pediatric17010006

**Published:** 2025-01-14

**Authors:** Leonardo Zoccante, Sara Sabaini, Erika Rigotti, Sophia Marlene Bonatti, Camilla Lintas, Marco Zaffanello

**Affiliations:** 1 Childhood, Adolescence, Families and Family Health Center, Azienda Ulss 9 Scaligera, 37122 Verona, Italy; leonardo.zoccante@aulss9.veneto.it (L.Z.); sara.psy.sabaini@gmail.com (S.S.); 2 Department of Paediatrics, Woman’s & Child’s, University Hospital of Verona, 37126 Verona, Italy; erika.rigotti@aovr.veneto.it; 3 Department of Neurosciences Biomedicine and Movement Sciences, Section of Physiology and Psychology, University of Verona, Strada Le Grazie 8, 37134 Verona, Italy; sophiamarlene.bonatti@univr.it; 4 Department of Mental Health, ULSS 9 Scaligera, 37122 Verona, Italy; camilla.lintas@aulss9.veneto.it; 5 Department of Surgery, Dentistry, Paediatrics and Gynaecology, University of Verona, 37126 Verona, Italy

**Keywords:** Asperger syndrome, autism spectrum disorder, level 1 ASD, level 1 autism, socialisation, social ability, Vineland score

## Abstract

Background/Objectives: Level 1 autism spectrum disorder (ASD) is a neurodevelopmental condition characterised by challenges in social and communication skills. Despite these difficulties, individuals with level 1 ASD often exhibit average intelligence and typical language development. Improving socialisation skills in this population requires tailored approaches that address their specific needs and include targeted strategies. This study aims to evaluate the effectiveness of a structured social skills training programme for adolescents and young adults with level 1 ASD. Methods: Participants diagnosed with level 1 ASD, regardless of gender, were consecutively recruited from an outpatient clinic. The intervention involved activities from the Social Skills, Autonomy, and Awareness Module, specifically designed for adolescents and young adults. Sessions were conducted fortnightly, lasting 1.5 to 3 h each, over 17 months. Adaptive behaviour was assessed using the Vineland Adaptive Behaviour Scales (VABS) at baseline and after completing the programme. Data were analysed with SPSS version 22.0 (SPSS Inc., Chicago, IL, USA). Statistical methods included automatic clustering to identify optimal clusters and Pearson’s Chi-square and Fisher’s exact tests to evaluate variable distributions among the clusters. Results: A total of 31 participants (77.4% female) with a mean age of 20.1 years (SD = 7.0) were included in the study. Two distinct clusters emerged. Cluster 1 (n = 8) had significantly higher mean ages and baseline Vineland II socialisation scores than Cluster 2 (n = 23). Both clusters demonstrated significant improvements in social skills following the intervention. Conclusions: This study highlights distinct profiles within individuals with level 1 ASD, showing a clear link between age and social skill development. The intervention improved social skills for most participants, regardless of the age at which treatment began. For some individuals, alternative or augmented treatment strategies may be necessary to achieve optimal results.

## 1. Introduction

In the DSM-5, level 1 autism spectrum disorder (ASD) is the mildest severity level of autism, encompassing individuals with less significant social and behavioural difficulties compared to the higher levels (level 2 and level 3). These individuals require minimal support for their difficulties but often have average or above-average language and cognitive abilities [1,2,3].

The current estimated prevalence of ASD is approximately 27.6 children per 1000, equivalent to roughly 1 in 36 children by the age of 8. Interestingly, ASD is 3.8 times more common in boys than girls, with rates of 43 per 1000 boys compared to 11.4 per 1000 girls [4].

Research has shown that level 1 ASD is often linked to differences in cognitive abilities and the presence of co-occurring conditions [5,6]. For instance, children with ASD may struggle with visuospatial skills, which are essential for understanding their surroundings, unlike typically developing children [7]. Additionally, children with non-verbal learning disabilities might excel in reading but face significant challenges with math concepts [8].

Encouraging children to join group activities or participate in playdates can offer them a structured and supportive way to practice social skills in real-world situations [8].

A review of the research compared various approaches to social skills training for young people with level 1 ASD and high-functioning autism, including traditional methods, cognitive-behavioural techniques, and programmes involving parents [9,10]. These interventions have significantly improved social abilities, particularly in fostering participation and mutual interaction in social settings [11].

Recently, there has been increasing interest in programmes designed to enhance social skills training (SST) for individuals with ASD. These programmes focus on improving social competence using a variety of strategies. SST has proven to be highly effective in helping individuals with ASD build communication and problem-solving skills [12]. A wide range of interventions has been developed to address the social challenges faced by children and adolescents with ASD. The choice of intervention is typically tailored to the individual’s age and level of functioning, ensuring it meets their specific needs and abilities [13,14].

To improve socialisation, tailored techniques grouped into distinct approaches have been employed [15,16]. Group-based interventions have demonstrated considerable efficacy in enhancing social skills [17]. Additionally, the integration of Behavioural Intervention Technologies (BITs), such as interactive software and therapeutic robots, has further expanded the potential of SST, offering promising new directions [17].

Despite these advancements, several challenges persist. The success of interventions often relies on customising them to meet an individual’s unique needs, incorporating strategies such as cognitive restructuring, emotional regulation techniques, and contingency management [9,18].

Additionally, most research has focused on school-aged children, leaving a significant gap in the understanding of how effective these interventions are for adolescents and young adults with ASD [8]. This underscores the importance of conducting targeted studies to evaluate the impact of SST in older age groups and to identify specific subgroups based on their initial characteristics. Such research could help develop more personalised and effective intervention strategies.

Providing personalised educational services tailored to the social needs of individuals with level 1 ASD is crucial [19]. Group-based social skills training has improved social abilities in adolescents and adults with ASD [20].

Research has highlighted the value of using behavioural techniques and innovative technologies in educational and clinical settings, stressing the importance of customising interventions to each person’s unique needs. SST programmes are widely recognised as an effective way to promote socialisation in individuals with ASD. However, it is important to note that responses to these programmes can vary from person to person.

### Aim of the Study

This study explores the effectiveness of a structured SST program tailored for adolescents and young adults with level 1 ASD. Unlike children, this group has received less attention in research. This study emphasises identifying distinct subgroups based on baseline characteristics to understand better how different individuals respond to the intervention.

## 2. Materials and Methods

### 2.1. Subjects

Participants with level 1 ASD, regardless of age or gender, were consecutively recruited from the outpatient clinic and voluntarily agreed to participate in the study. To be included, participants needed a confirmed diagnosis of level 1 ASD with no coexisting medical or psychiatric conditions. Individuals with any comorbidities were excluded.

Most participants received support from their families, who played a key role in encouraging skill development beyond the structured SST sessions. While detailed data on the families’ educational or socioeconomic backgrounds were not systematically collected, informal observations indicated a wide range of parental involvement. Some parents actively reinforced the skills taught during the program, while others participated less due to personal or situational factors.

This study adhered to the principles outlined in the Declaration of Helsinki. It was approved by the Institutional Review Board (Ethics Committee) of the University Hospital of Verona (CESC 2243 for the Paediatric Clinic and CESC 2242 for the Child and Adolescent Neuropsychiatry Outpatient Clinics) on December 10, 2019. Informed consent was obtained from all participants and their legal guardians before the start of the study.

### 2.2. Behavioural Intervention

The proposed activities, centred on the Social Skills, Autonomy, and Awareness Module, are essential components of interventions aimed at improving the quality of life and overall functioning of individuals, particularly those with ASD [21,22,23,24].

From May 2021 to February 2022, the adolescent group participated in practical sessions held every two weeks. This schedule was designed to balance the program’s intensity with the participants’ ability to process and retain information between sessions. The activities focused on developing socialisation, autonomy, and self-awareness through engaging, hands-on experiences. For instance, participants explored the local community to practice social interactions in real-life settings. They also participated in cooking workshops, where they learned to prepare simple meals, promoting teamwork and practical life skills. Additionally, role-playing exercises allowed them to rehearse social situations in a structured environment, enhancing their communication and problem-solving abilities.

The young adult group engaged in structured discussions on topics relevant to their everyday lives, such as employment, relationships, and independence. These discussions were complemented by role-playing activities that simulated real-life scenarios. Practical outings were organised to practice essential daily living skills, such as shopping, cooking, and tidying up. Participants created a WhatsApp group to coordinate schedules and share reminders to facilitate communication and engagement between sessions.

In total, 13 sessions were conducted between May 2021 and February 2022. Most sessions lasted about 1.5 h, but some practical sessions extended to 3 h to accommodate more complex activities. This flexible approach allowed the sessions to adapt to the needs of the participants, ensuring a meaningful and productive experience.

### 2.3. Vineland Test

The VABS (Vineland Adaptive Behaviour Scales) is a diagnostic tool for assessing adaptive behaviour and various developmental disabilities [25]. These scales measure how well an individual functions in everyday life and help identify areas of difficulty in adaptive behaviour, focusing on four key domains: motor skills, communication, daily living skills, and socialisation [26]. The Vineland ABS has been especially useful in supporting ASD diagnoses with other diagnostic methods [25]. This tool highlights specific deficit areas and tracks progress in social and daily living skills over time [25,27]. The assessment was conducted at the beginning of the study and repeated during the follow-up to evaluate the effectiveness of the Social Skills, Autonomy, and Awareness Module.

### 2.4. Statistical Analysis

Data were exported from an online questionnaire created using Google Forms and saved in XLS file format. They were then entered into a Microsoft^®^ Excel^®^ database for Windows 11 (accessed on 30 September 2021). Statistical analysis was performed using SPSS version 22.0 for Windows (SPSS Inc., Chicago, IL, USA).

We used the one-sample Kolmogorov–Smirnov test to assess the normality of data distribution.

We performed a clustering analysis to identify distinct subgroups of participants based on baseline characteristics. This approach was chosen to address our hypothesis that adolescents and young adults with level 1 ASD may exhibit heterogeneous responses to the SST programme. Identifying these subgroups provides insights into differential responses to the intervention, helping to determine which baseline characteristics may influence its effectiveness.

For clustering analysis, automatic clustering techniques with the Bayesian Information Criterion (BIC) were applied to determine the optimal number of clusters within the dataset.

We used Pearson’s Chi-square test for categorical data and two-tailed t-tests for continuous data with a normal distribution to compare differences in cluster variables. For data that were not normally distributed, we used the Mann–Whitney test.

## 3. Results

Outpatient enrolment resulted in 31 patients (24 females; 77.4%) with a mean age (SD) of 20.1 (7.0) years. Demographic characteristics of participants by age group are reported in Table 1.

The two-cluster model (Table 2) proved the most efficient, with the lowest BIC score and a satisfactory distance measure ratio of 1.913. This indicates a clear separation between clusters without unnecessary complexity.

Based on the Bayesian Information Criterion (BIC) and the distance ratio of 1.913 for the two-cluster solution, the clustering analysis suggests a reliable separation between clusters. This is consistent with a silhouette measure that likely exceeds the 0.5 threshold commonly used to assess clustering quality.

The analysis in Table 3 compares two groups (Cluster 1, with eight participants, and Cluster 2, with twenty-three participants). Pearson’s Chi-square test was used to explore gender differences, while a two-tailed *t*-test was applied to compare age differences and scores on the Vineland subscales. The Mann–Whitney U test was used for the total Vineland scores, which were not normally distributed. No significant difference in gender distribution was found between the two groups (*p* = 0.600).

Initially, the average age in Cluster 1 was significantly higher than in Cluster 2 (*p* = 0.004). This trend persisted at follow-up, with the mean age of Cluster 1 continuing to be significantly higher than that of Cluster 2 (*p* = 0.001).

Regarding the Vineland scores, Cluster 1 had a significantly higher mean score at enrolment than Cluster 2 (*p* < 0.001). This trend continued at follow-up, with Cluster 1 maintaining a significantly higher mean score than Cluster 2 (*p* < 0.001). The change in scores from enrolment to follow-up for Cluster 1 and Cluster 2 showed no significant difference (*p* = 0.052 for Cluster 1 and p = 0.354 for Cluster 2).

For the Vineland communication scores, the difference between the two groups at enrolment was highly significant (*p* < 0.001), indicating that Cluster 1 initially had much stronger communication skills than Cluster 2. This significant difference persisted at follow-up (*p* < 0.001). However, no significant change was observed within Cluster 1 from enrolment to follow-up (*p* = 0.134), nor within Cluster 2 (*p* = 0.889).

For daily living skills, the difference between the groups was highly significant at both enrolment (*p* < 0.001) and follow-up (*p* < 0.001). The mean score for daily living skills in Cluster 1 increased at follow-up, but no significant internal changes were found in either Cluster 1 (*p* = 0.123) or Cluster 2 (*p* = 0.168).

Lastly, the Vineland socialisation scores at both enrolment and follow-up established a highly significant difference between the two groups (*p* < 0.001). At follow-up, the mean scores for both groups increased, demonstrating significant improvements over time (Cluster 1, *p* = 0.036; Cluster 2, *p* = 0.009).

**Figure 1:**
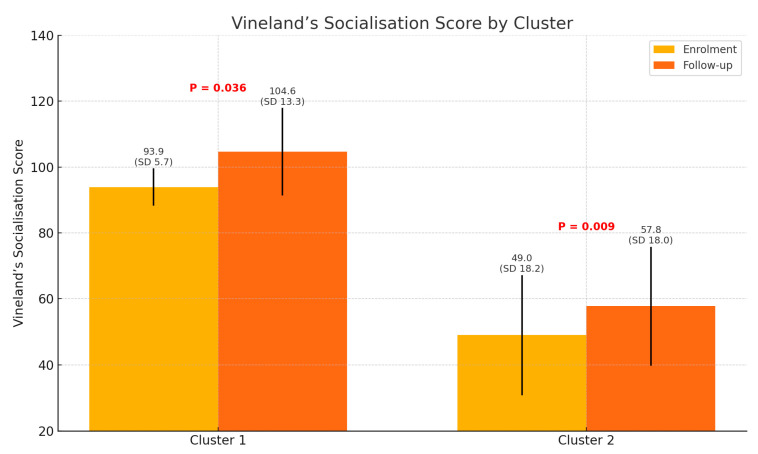
The bar chart displays the mean (SD, standard deviation) Vineland scale scores for socialisation in Clusters 1 and 2, measured at enrolment and follow-up.

Figure 1 shows that at follow-up (represented by the green bar), the mean score in Cluster 1 demonstrated a statistically significant increase compared to enrolment (indicated by the red bar; *p* = 0.036), highlighting a meaningful improvement in socialisation for Cluster 1. Likewise, at follow-up, Cluster 2 also exhibited an increase in its mean score compared to enrolment (*p* = 0.009), suggesting an enhancement in socialisation. However, its scores remained lower than those of Cluster 1.

**Figure 2:**
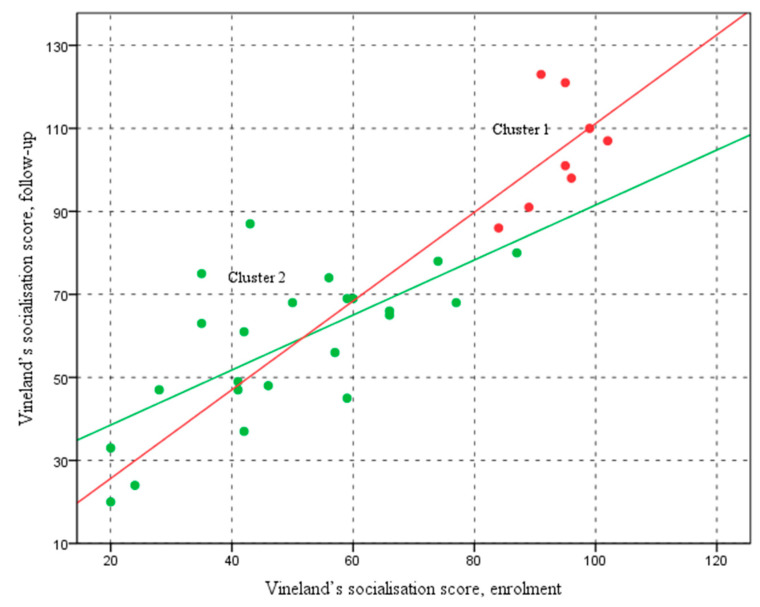
The graph illustrates the relationship between the Vineland socialisation scores at two different time points: the enrolment assessment (on the horizontal axis) and the follow-up assessment (on the vertical axis). Each point on the graph represents a participant, with different colours indicating their cluster membership. This visual representation helps to show how socialisation scores have changed over time for each participant, with colour coding highlighting the distinct clusters.

Figure 2 shows that Cluster 1, represented in red, consists of participants whose scores remain relatively stable at higher levels, as indicated by their positioning within the higher range of enrolment and follow-up scores. The linear regression line for Cluster 1 (R^2^ = 0.213, y = 4.29 + 1.07x) indicates a moderate positive relationship between enrolment and follow-up scores, with a 1.07 slope suggesting a gradual increase in scores over time.

In contrast, Cluster 2, shown in green, exhibits a wider distribution of scores, with enrolment and follow-up scores spanning a broader range. The regression line for Cluster 2 (R^2^ = 0.448, y = 25.3 + 0.66x) demonstrates a steeper slope, indicating a more pronounced improvement in socialisation over time than Cluster 1. This suggests that the participants in Cluster 2 experienced greater variability in their scores but showed a more substantial improvement from enrolment to follow-up, as reflected by the higher R^2^ value.

**Figure 3:**
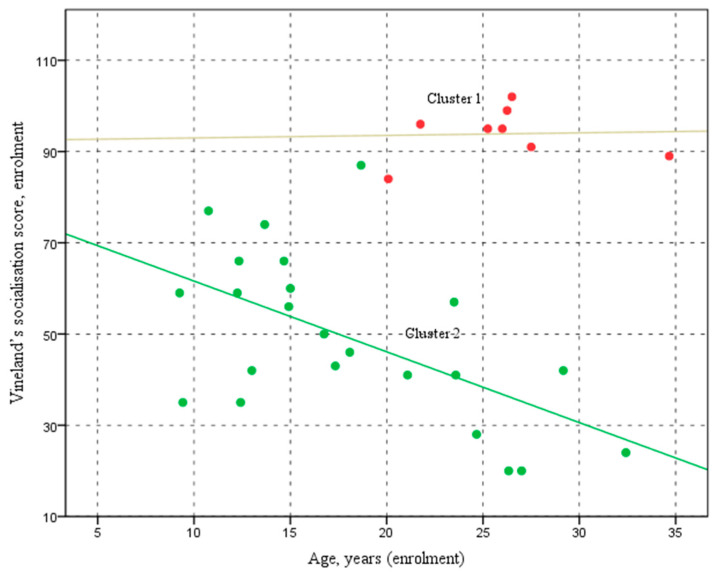
The scatter plot illustrates the relationship between enrolment age (on the horizontal axis) and the Vineland socialisation score at enrolment (on the vertical axis) for both the Cluster 1 and Cluster 2 groups. Each point represents an individual participant, with Cluster 1 and 2 distinguished by different colours. The plot highlights patterns or trends in how age at enrolment correlates with socialisation scores, allowing for a visual comparison between the two clusters.

In Figure 3, the scatter plot displays the relationship between the two groups enrolment age (horizontal axis) and Vineland socialisation scores at enrolment (vertical axis). Participants in Cluster 1 (represented by the red points) are between approximately 20 and 35 years old, with socialisation scores ranging from 80 to 110. For Cluster 1 (red spots), the analysis shows an almost flat relationship between age and socialisation scores (y = 92.42 + 0.06x, R^2^ = 0.002), indicating that age does not significantly affect socialisation scores in this group.

In contrast, participants in Cluster 2 (shown by the green points) span a broader age range, from about 7 to 35 years old, with socialisation scores varying significantly from around 20 to 90. Conversely, for Cluster 2 (green spots), the analysis reveals a clear downward trend in socialisation scores with increasing age (y = 77.12 − 1.55x, R^2^ = 0.322), suggesting that age significantly impacts the decrease in scores for this group.

**Figure 4:**
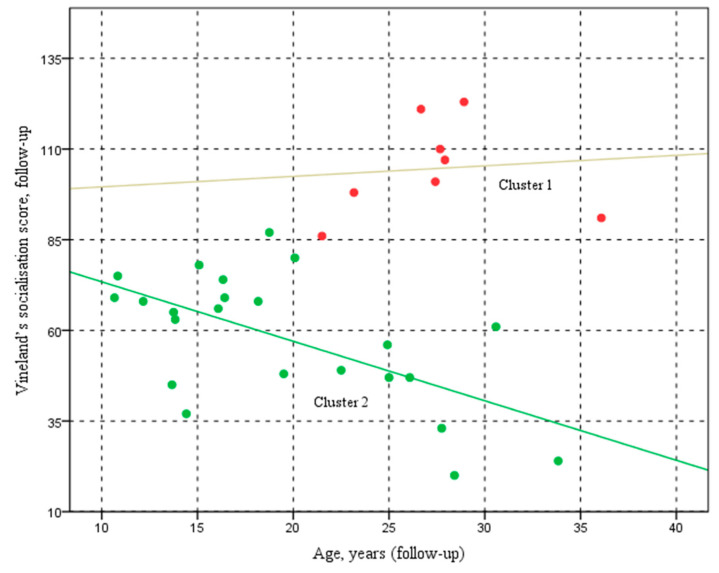
The scatter plot illustrates the relationship between age at follow-up (horizontal axis) and the Vineland socialisation score at follow-up (vertical axis) for the two groups, Cluster 1 and Cluster 2.

In Figure 4, the participants in Cluster 1 (red points) are aged between 20 and 37 years, with follow-up socialisation scores ranging from 85 to 120. The trend line for Cluster 1 is almost horizontal. The regression line (y = 96.67 + 0.29x, R^2^ = 0.009) suggests an almost flat relationship between age and socialisation scores during follow-up. This indicates that, although there is a slight tendency for socialisation scores to improve with increasing age, the variability among the data is very high, as highlighted by the low R^2^ value.

In contrast, participants in Cluster 2 (green spots) are aged approximately 10 to 35 years, and their follow-up socialisation scores vary widely, falling between 20 and 90. The regression line (y = 89.79 − 1.64x, R^2^ = 0.368) shows a more evident downward trend in socialisation scores with age, suggesting that older participants tend to have lower socialisation scores. The R^2^ value, although not very high, is still significant and indicates a certain degree of correlation between age and scores.

**Figure 5:**
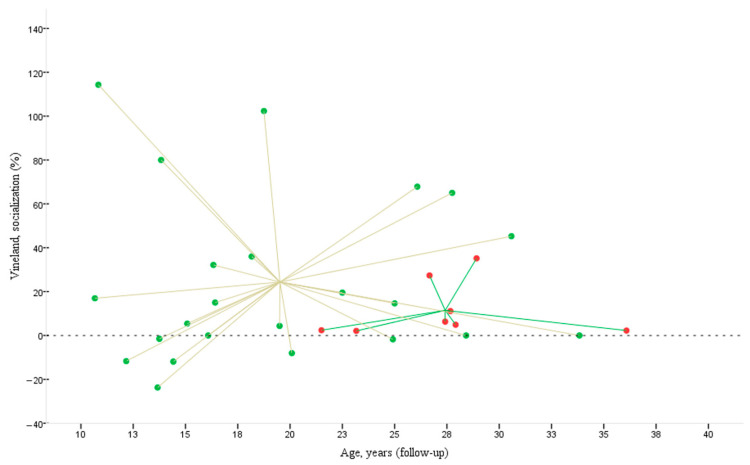
The figure illustrates the percentage change in Vineland socialisation scores (%) about age (in years) at follow-up for two groups: Cluster 1 and Cluster 2. Age is plotted along the horizontal axis, while the Vineland socialisation scores are displayed vertically. Each point on the graph represents a participant, with Cluster 1 marked in red and Cluster 2 in green. The lines connecting the points converge at a point representing each cluster’s centroid.

In Figure 5, Cluster 1 (marked by red points) consists of older individuals whose socialisation scores, representing a percentage change, remain slightly above zero, indicating only a marginal increase in their socialisation. In contrast, Cluster 2 (represented by green points) includes individuals with much greater variability in their Vineland socialisation change (%). Some individuals in this cluster achieve very high scores, while others show markedly negative scores (e.g., −4; −12.9%), indicating a consistent decline in socialisation.

## 4. Discussion

This study evaluated the effectiveness of the Social Skills, Autonomy, and Awareness Module in individuals of various ages with level 1 ASD. The analysis identified two distinct groups (clusters) with differing clinical profiles.

The first group, comprising eight participants, had higher Vineland socialisation scores at both enrolment (93.9 ± 5.7) and follow-up (104.6 ± 13.3). Conversely, the second group, with 23 participants, demonstrated a broader range of Vineland socialisation scores, starting lower at enrolment (49.0 ± 18.2) and follow-up (57.8 ± 18.0). Notably, 12.9% of participants in this second group—particularly those under 20—showed an unexpected decline in socialisation scores. Overall, the change in socialisation scores from enrolment to follow-up was statistically significant in both groups (*p* = 0.036 for Cluster 1 and *p* = 0.009 for Cluster 2). This finding highlights the importance of age and socialisation experiences in fostering socialisation among individuals with level 1 ASD.

In the first group, the change in Vineland scores from enrolment (105.4 ± 13.4) to follow-up (110.9 ± 14.8) was not statistically significant (*p* = 0.052). Similarly, the second group showed a more modest change in scores from enrolment (64.8 ± 14.1) to follow-up (66.6 ± 15.5), which was also not statistically significant (*p* = 0.354).

According to Chen et al., our findings suggest the existence of distinct profiles among level 1 ASD subjects, potentially shaped by varying life experiences [28], therapies undertaken, and individual factors related to neuropsychiatric comorbidities. When individuals were grouped into two clusters based on their socialisation at enrolment, both clusters demonstrated improvements, albeit with some exceptions noted among younger participants (≤20 years old). Notably, intellectual disability and language abilities emerged as critical variables influencing the socialisation of children with ASD [29].

In our study, adolescents and young adults participated in a structured exploratory training programme that met biweekly, with sessions ranging from 1.5 to 3 h. These sessions incorporated various practical activities. Furthermore, the young adults established a social media group to enhance communication and interactions. The utilisation of social networks can indeed assist individuals with ASD in overcoming communication barriers [30]. Despite the challenges they encounter, adolescents with ASD are eager to forge social connections [30]. While there is limited high-quality evidence on the role of social media for individuals with ASD [31], existing reports are promising, suggesting that social media-based approaches could effectively facilitate behavioural changes in this population [32].

Practical guidance is essential to help individuals with ASD navigate social networks effectively [30]. Our group of young adults may have improved their socialisation through spontaneous engagement with social media, a phenomenon that aligns with findings in the existing literature. The challenges observed in younger participants are consistent with developmental patterns and tend to diminish with age [33].

The first group, comprising individuals with level 1 ASD, was generally older than the second group and consistently achieved significantly higher scores across various Vineland subscales at enrolment and follow-up. These results suggest that this group may have better adaptive skills and greater daily functioning at baseline, as supported by prior studies [25]. Social skills training (SST) has been shown to enhance social competencies in adults with ASD [34], which might explain the continued improvement in Vineland scores observed in the first group at follow-up. Although this improvement approached statistical significance, it highlights the potential for ongoing learning and progress in older individuals. However, these findings should be interpreted cautiously due to the moderate quality of existing evidence, which may have exaggerated the observed effects [35]. Further research must validate these results and understand the underlying factors contributing to these outcomes [34].

The second group, predominantly younger, began with lower initial scores compared to the first group, as demonstrated by their significantly lower enrolment Vineland scores, a finding consistent with previous studies [36]. Nevertheless, this group showed notable improvements in Vineland socialisation scores from enrolment to follow-up. This aligns with evidence from a systematic review indicating that intervention groups receiving occupational therapy tend to improve their overall socialisation [37]. Additionally, the PEERS programme has been shown to effectively enhance social skills among autistic adolescents by focusing on improving social knowledge [38].

The lower initial competence of younger participants in the Social Skills, Autonomy, and Awareness Module can likely be attributed to various factors. These include developmental mismatches between the programme activities and participants’ needs, challenges in engaging with the material [39], and the overall quality and suitability of the programme’s implementation. Increasingly, research emphasises the importance of tailoring interventions to the specific needs of different age groups and individuals [40]. While Social Skills Group Programmes (SSGPs) have demonstrated potential in improving socialisation for individuals with ASD, addressing methodological and implementation challenges is crucial to achieving more reliable and valid outcomes [34]. In this study, the activities for the younger group were meticulously designed using evidence-based interventions to promote socialisation, autonomy, and self-awareness, aiming to bridge these gaps effectively [24,26].

Both groups demonstrated a significant improvement in socialisation, suggesting that this area is particularly responsive to therapeutic interventions. Group-based training, when accessible, can be highly beneficial as it provides structured opportunities for social interaction. Therapists may also guide individuals with level 1 ASD to leverage their unique strengths to foster more socialisation opportunities [12]. The first group, which started with higher scores, continued to show meaningful improvements, maintaining their advantage over the second group. The positive relationship observed between the enrolment and follow-up socialisation scores in both groups indicates that individuals with stronger initial skills are likely to sustain or further develop these abilities over time.

Although younger individuals with level 1 ASD typically show more significant potential for socialisation improvements through interventions, the approach used in this case may not have been sufficiently practical in significantly advancing their communication and daily living skills.

In the first group, there is a slight average decline in communication scores and a corresponding increase in daily living skills scores from enrolment to follow-up. While these changes are noticeable, they do not reach statistical significance. Similarly, the second group exhibits a minor average decline in daily living skills over the same period, which is also not statistically significant.

This observation is consistent with prior research highlighting that individuals with ASD often demonstrate slower progress in daily living skills compared to their peers with non-spectrum diagnoses, likely due to more pronounced challenges in non-verbal cognition [41]. Cognitive deficits in individuals with ASD persist from childhood into adulthood [42]. Regression analyses further indicate that the severity of ASD is a significant predictor of outcomes in socialisation and daily living skills [43]. Cognitive deficits in individuals with ASD persist from childhood into adulthood [42], with older adults frequently failing to reach typical levels of social functioning despite some symptom improvements over time [44]. These findings highlight the complex relationship between developmental delays, cognitive difficulties, and the necessity for tailored interventions to address the varied adaptive skill profiles within the ASD population.

This study has some limitations, most notably the small sample size, which did not allow for a balanced distribution of participants between the two clusters. The smaller size of Cluster 1 likely reflects the characteristics of its members, who were grouped based on their adaptive functioning (as measured by Vineland scores) and demographic factors such as age and gender. Cluster 1 represents a subgroup with higher adaptive functioning, as evidenced by their consistently higher Vineland scores at both enrolment and follow-up and higher average age. Although this imbalance was unexpected, it provided valuable insights into the diverse profiles of individuals with level 1 ASD included in the study.

In our study, most participants were female, which contrasts with the male predominance typically observed in ASD cases [45]. This gender imbalance was not due to intentional selection bias but reflected the composition of the patient population attending our clinic during recruitment. Our inclusion criteria aimed to consecutively enrol patients with level 1 ASD, regardless of gender or age, as long as they had no medical or psychiatric comorbidities. Additionally, the higher proportion of females in our sample may be partly explained by referral and diagnostic biases often associated with ASD. Females with ASD, especially those with level 1 ASD, tend to present with behavioural and social profiles that differ from those of males, which can influence diagnosis and referral patterns [45].

While the Vineland Adaptive Behaviour Scales (VABS) offer standardised and reliable measures of adaptive behaviour, their quantitative approach may not fully capture the nuanced, qualitative aspects of individual progress [25]. Future research should consider incorporating qualitative assessments alongside standardised measures to provide a more comprehensive understanding of intervention outcomes.

Another potential limitation of this study is the variability in parental involvement, which was not systematically assessed [46]. Future research should incorporate a more detailed evaluation of parental engagement and other family-related factors to understand better their influence on the effectiveness of social skills training (SST) programmes.

One of this study’s notable strengths is the broad age range of participants, which enabled an assessment of the intervention’s effectiveness across different life stages and varying levels of socialisation at enrolment. Significantly, the intervention improved socialisation in over half of the individuals with level 1 ASD, regardless of the age at which treatment commenced. However, the findings also highlight that not all participants benefited from the intervention, with some showing no improvement or even a decline in socialisation. This underscores the need for further research to refine and tailor these programmes to individual needs.

## 5. Conclusions

This study offers valuable insights into the effectiveness of the Social Skills, Autonomy, and Awareness Module for individuals with level 1 ASD, highlighting how age and social experience shape distinct adaptive profiles. Both groups improved socialisation, underscoring the importance of tailoring interventions to individual developmental stages and needs. The findings suggest that older individuals with level 1 ASD may benefit from their greater social experience and adaptive capacities, as reflected in their higher baseline and follow-up scores. However, younger participants showed more variable responses, with some displaying limited progress or declines, pointing to the need for age-specific modifications in intervention strategies.

Despite its limitations, including the small sample size and potential gender bias, this study emphasises the importance of structured, evidence-based interventions for improving socialisation. Future research should incorporate qualitative measures, assess the influence of family dynamics, and explore innovative approaches, such as social media-based interventions, to enhance and expand the effectiveness of such programmes.

## Figures and Tables

**Table 1:** Demographic characteristics of participants by age group (adolescents and young adults).

Variable	Adolescents (n = 13)	Young Adults (n = 18)
Age, mean (SD)	13.2 (2.5)	25.1 (4.4)
Age range (years)	9.3–17.3	18.1–34.7
Sex, n (%)		
Male	2 (28.6%)	5 (45.8%)
Female	11 (71.4%)	13 (54.2%)

**Table 2:** The statistical analysis presents the results of automatic clustering, which employed the Bayesian Information Criterion (BIC) to determine the optimal number of clusters within the dataset. The variables analysed are outlined in Table 2 for both the enrolment and follow-up stages. The change ratios correspond to the solution that identified two clusters, offering insights into how participants responded to the intervention.

Number of Clusters	Bayesian Criteria of Schwarz (BIC) Criteria	Change in BIC	Change Ratio	Distance Measures
1	315.066			
2	298.592	−16.475	1.000	1.913
3	324.390	25.798	−1.566	1.621
4	367.935	43.545	−2.643	2.733

**Table 3:** The table presents the statistical results for two groups of individuals with autism, assessed at different time points (enrolment and follow-up). It includes their ages and scores from the Vineland scale, focusing on socialisation, communication, and daily living skills. The table also compares the two groups and highlights the *p*-value figures used to assess the statistical significance of the results.

Variable	Cluster 1 (n.8)	-	-	Cluster 2 (n.23)		Pearson’s Chi-Squared (*p*-Value; Cramér’s V)	Kolmogorov-Smirnov Test	*t*-Test
Gender, females (%)	6 (75.0)	-	-	18 (78.3)		0.036 (0.600; 0.849)	-	-
	Mean (SD)	95% C.I.	*p*-value (paired samples *t*-test)	Mean (SD)	95% C.I.	*p*-value (paired samples *t*-test)	*p*-value, two taled (test statistic)	*p*-value, two-tailed (t-statistic)
Age (years), [range], enrolment	26.0 (4.3) [20.1–34.7]	22.4–29.6	-	18.1 (6.6) [9.3–32.4]	15.2–21.0	-	0.200 (0.123)	0.004 (3.120)
Age (years), follow-up	27.4 (4.3)	23.8–31.0	-	19.5 (6.7)	16.6–22.4	-	0.200 (0.123)	0.001 (3.120)
Vineland score, enrolment	105.4 (13.4)	94.1–116.6	0.052	64.8 (14.1)	58.7–70.9	0.354	0.045 (0.158)	<0.001 *
Vineland score, follow-up	110.9 (14.8)	98.5–123.2		66.6 (15.5)	59.9–73.3		0.014 (0.092)	<0.001 *
Vineland communication score, enrolment	96.1 (13.4)	84.9–107.3	0.134	60.1 (20.9)	51.1–69.2	0.889	0.200 (0.102)	<0.001 (4.530)
Vineland communication score, follow-up	92.8 (11.5)	83.1–102.4		60.5 (19.6)	52.0–69.0		0.200 (0.092)	<0.001 (4.365)
Vineland’s daily living skills score, enrolment	109.1 (8.9)	101.7–116.5	0.123	66.3 (29.3)	46.6–75.9	0.168	0.200 (0.110)	<0.001 (5.244)
Vineland daily living skills score, follow-up	114.9 (13.9)	103.3–126.5		63.8 (20.6)	66.1–87.8		0.166 (0.134)	<0.001 (6.480)
Vineland’s socialisation score, enrolment	93.9 (5.7)	89.1–98.7	0.036	49.0 (18.2)	41.2–56.9	0.009	0.200 (0.111)	<0.001 (6.789)
Vineland’s socialisation score, follow-up	104.6 (13.3)	93.5–115.7		57.8 (18.0)	50.0–79.7		0.200 (0.094)	<0.001 (6.726)

Legend: SD, standard deviations; 95% C.I., 95% confidence intervals; * Mann–Whitney test. The light blue indicates a significance level >0.05 and <0.1; green highlights statistically significant results with *p* < 0.05.

## Data Availability

Data are unavailable due to privacy and ethical restrictions.
